# A new hope for pulmonary hypoplasia: amniotic fluid stem cell extracellular vesicles?

**DOI:** 10.20517/evcna.2025.25

**Published:** 2025-07-10

**Authors:** Ishara Atukorala, Lisa Hui

**Affiliations:** ^1^Department of Obstetrics, Gynaecology and Newborn Health, Melbourne Medical School, The University of Melbourne, Heidelberg 3084, Australia.; ^2^Mercy Perinatal, Mercy Hospital for Women, Heidelberg 3084, Australia.; ^3^Northern Health, Epping 3076, Australia.; ^4^Murdoch Children’s Research Institute, Parkville 3052, Australia.

**Keywords:** Extracellular vesicles, pulmonary hypoplasia, inflammation, amniotic fluid stem cells

## Abstract

In a recent study, Antounians and colleagues (2024) investigated the potential of amniotic fluid stem cell-derived extracellular vesicles (AFSC-EVs) as a therapeutic intervention for pulmonary hypoplasia resulting from congenital diaphragmatic hernia (CDH). They demonstrated the pronounced inflammatory signature of the fetal hypoplastic lungs, marked by heightened macrophage density in a CDH rat model. Intra-amniotic AFSC-EV administration mitigated the inflammatory process and enhanced fetal lung development by promoting branching morphogenesis and epithelial maturation. These findings add to a growing body of preclinical and clinical evidence supporting the therapeutic potential of AFSC-EVs for inflammation-driven pathologies.

## MAIN TEXT

Congenital diaphragmatic hernia (CDH) is a severe developmental condition resulting from the incomplete closure of the diaphragm during fetal development. This defect allows abdominal organs to herniate into the thoracic cavity, compressing the fetal lungs. This anatomic distortion disrupts normal lung development. As a result, the fetus develops pulmonary hypoplasia^[1]^, a condition characterised by reduced branching morphogenesis, fewer alveoli, and impaired vascularisation. Pulmonary hypoplasia remains a leading cause of morbidity and mortality in affected children, as the underdeveloped lungs struggle to support postnatal respiration^[2]^. Despite advances in fetal interventions^[3]^, newborn critical care and postnatal surgery, pulmonary hypertension and reduced lung function remain problems in survivors^[4]^. Therefore, interventions promoting normal lung development before birth are a pressing clinical need.

Building on earlier work demonstrating the regenerative capabilities of amniotic fluid stem cell-derived extracellular vesicles (AFSC-EVs) in experimental models of lung hypoplasia^[5-7]^, Antounians *et al*. explored the molecular and cellular changes induced by AFSC-EV therapy in fetal hypoplastic lungs^[8]^. Choosing a well-characterised CDH model in this study enhances the clinical relevance of the findings, as the treatment was tested within the intricate physiological complexities of a living organism. Their approach included the administration of AFSC-EVs via intra-amniotic injection. The treatment effectively targeted the fetal lungs, enhanced branching morphogenesis, and promoted epithelial cell differentiation, supporting both structural and functional maturation of lungs. Using single-nucleus RNA sequencing (snRNA-seq), they identified a multilineage inflammatory signature in the untreated hypoplastic lungs, with a significant presence of macrophages in the inflamed tissue. The machine learning approach revealed that most of these were tissue-resident alveolar macrophages (about 77%), with a proportion of monocyte- and bone marrow-derived recruited macrophages. They displayed a pro-inflammatory phenotype, evidenced by high expression of inflammatory markers such as TNFα, Lcn2, Il1b, Ccl3/4, and Cxcl1. This inflammatory signature was effectively improved by AFSC-EV treatment, downregulating the macrophage signature.

This study advances our understanding of CDH-associated lung pathology and highlights the broader promise of AFSC-EV therapy in inflammation-driven diseases. Researchers have demonstrated the strong anti-inflammatory and regenerative properties of AFSC-EVs in numerous studies^[9]^. As a naturally derived cell-free therapeutic, AFSC-EVs offer advantages such as biocompatibility and minimal toxicity. These findings resonate with a broader shift towards EV-based therapies, which provide cell-free approaches to harness the paracrine benefits of stem cells^[10]^. EV-based therapies are also gaining popularity in clinical settings. A search of the international clinical trials registry for interventions involving “extracellular vesicles” yielded over 150 registered clinical trials. Stem cell-derived EVs have been investigated in humans for conditions such as COVID-19-induced respiratory failure^[11]^ and chronic wound healing^[12]^, with reported safety and efficacy. Additionally, amniotic fluid-derived EVs have been used to treat prolonged respiratory symptoms in COVID-19 patients, further supporting their translational potential and safety of amniotic fluid-derived therapeutics in human subjects^[13,14]^.

Currently, the main prenatal therapy for severe CDH is fetoscopic endoluminal tracheal occlusion (FETO)^[15]^, which stimulates lung growth by temporarily blocking the fetal trachea. This approach has shown improved survival from ~15% to ~40% in a randomised trial^[3]^. But FETO is highly invasive and carries risks such as preterm rupture of membranes and preterm birth^[15,16]^. In comparison, AFSC-EVs provide a less invasive and potentially safer cell-free alternative to improve fetal lung regeneration.

Intra-amniotic delivery is clinically appealing because it leverages a routine prenatal procedure (amniocentesis) to deliver therapy directly to the fetus. The timing of intervention in this discussed rat model was during mid-gestation (analogous to the canalicular stage of lung development). Translating this approach to humans would likely target the second trimester, when fetal lung branching is most active, aligning with the gestation period when amniocentesis is typically performed. Importantly, literature^[17-19]^ supports the feasibility of the intra-amniotic route for therapeutic delivery, further underscoring its potential for fetal intervention therapy.

The utilisation of snRNA-seq in this study is particularly noteworthy, as it enables the precise characterisation of gene expression profiles within complex tissues. This technique minimises cell disruption during isolation, leading to a more accurate representation of tissue-resident cell populations^[20]^. However, it is not without caveats: nuclear RNA abundance does not always reflect cytoplasmic transcript levels, which could potentially lead to misinterpretations of the types and quantities of active RNA that contribute to cellular functions. In contrast, single-cell RNA-seq (scRNA-seq) offers more comprehensive transcriptome coverage but may introduce bias due to its dependence on cell viability^[21]^.

A key strength of this research is the use of good manufacturing practice (GMP)-grade AFSCs for EV isolation. The implementation of GMP standards ensures the reproducibility, safety, and scalability of therapeutic products, an essential step towards clinical application. Further refining methods for manufacturing GMP-grade EVs would enhance the translational impact of this genre of research. Progress has been made in this aspect with the use of automated bioreactors to scale up EV production^[22]^, with significant reductions in manufacturing costs^[23]^. Scalable EV isolation methods, such as tangential flow filtration, have shown promise; however, they are yet to be optimised for pairing with other techniques, like size-exclusion chromatography, to isolate specific EV subtypes^[24]^.

Despite the promising findings, there are still gaps that warrant further investigation. The precise mechanisms through which AFSC-EVs exert their therapeutic effects remain to be fully understood. In the current study, it is unclear which macrophage subsets are most affected by AFSC-EV therapy. As the authors acknowledged, future studies could benefit from integrating complementary techniques such as spatial transcriptomics and fate mapping to elucidate the complex inflammatory landscape of hypoplastic lungs. Moreover, elucidating the key anti-inflammatory and regenerative components of AFSC-EVs will be especially important. Studies indicate that specific microRNAs in the EV cargo, such as members of the miR-17~92 cluster^[7]^ and miR-93-5p^[25]^, can enhance fetal lung branching morphogenesis. The anti-inflammatory properties of stem cell EVs have also been attributed to proteins such as TGF-β and VEGF, among others^[26]^.

Additionally, the long-term safety and efficacy of intra-amniotic AFSC-EV administration should be explored in larger animal models and, eventually, in appropriately powered clinical trials. Particularly, the potential immunogenicity and off-target effects of AFSC-EVs need consideration. As a cell-free product, AFSC-EVs are less likely to trigger direct immune rejection^[27]^. However, the biodistribution of administered EVs requires a thorough understanding. While intra-amniotic injection successfully delivers EVs to the fetal lungs, vesicles may also be absorbed by other fetal tissues or enter the maternal circulation. It will be critical to verify that AFSC-EV therapy does not produce unintended off-target effects in distant sites.

In conclusion, the study by Antounians *et al.* provides compelling evidence of the potential of AFSC-EVs as a novel therapeutic strategy for fetal pulmonary hypoplasia associated with CDH^[8]^. By combining a robust experimental design with advanced molecular profiling, this study underscores the feasibility and promise of AFSC-EVs as a cell-free approach for fetal lung hypoplasia and, potentially, other congenital or perinatal inflammatory conditions. With future studies exploring molecular mechanisms behind these therapies and refining intra-amniotic delivery protocols, the field is poised to make significant strides towards innovative fetal interventions that could transform the prognosis for infants and families affected by CDH and other congenital conditions.

## DECLARATIONS

### Copyright

© The Author(s) 2025.
